# Clinical characteristic and pathogenesis of tumor-induced acute pancreatitis: a predictive model

**DOI:** 10.1186/s12876-022-02501-9

**Published:** 2022-09-15

**Authors:** Linlin Zheng, Ping Zhao, Xiaoqian Peng, Yunhui Zhou, Yichen Bao, Yuling Sun, Lin Zhou

**Affiliations:** 1 Department of Gastroenterology, The First Affiliated Hospital of Zhengzhou University, No.1, East Jianshe Road, Zhengzhou, 450052 China; 2 Department of Gastroenterology, Jiangsu Provincial Hospital, No.300, Guangzhou Road, Nanjing, 210029 China; 3 Department of Hepatobiliary and Pancreatic Surgery, The First Affiliated Hospital of Zhengzhou University, No.1, East Jianshe Road, Zhengzhou, 450052 China

**Keywords:** Tumor-induced acute pancreatitis, Characteristics, Pathogenesis, Predictor, Machine learning

## Abstract

**Background:**

The aim of our study was to investigate the clinical characteristics and pathogenesis of tumor-induced acute pancreatitis (AP), and to develop a reliable prediction model of the clinical features to guide the diagnosis and treatment.

**Methods:**

Patients with AP between January 2013 and December 2021 were enrolled in the study and were subdivided into the tumor group and the non-tumor group. The tumor group was subdivided into three groups based on the primary sites. Characteristic parameters, laboratory and imaging results were compared between groups. Least absolute shrinkage and selection operator regression model, XGBoost and random forest model were used to select the predictors associated with tumor-induced AP. Logistic regression analysis was used to validate the performance of the selected predictors and a nomogram was established to provide individualized probability of a tumor origin for AP.

**Results:**

A total amount of 8970 patients were admitted for AP during the study period, and 8637 AP patients were enrolled in the study. Of these, 100 cases (1.16%) were tumor-induced AP. The tumor group was significantly older than the non-tumor group (t = 6.050, *p* = 0.000). Mild AP was observed in 90 cases, moderate AP in 9 cases and severe AP in one case. Tumors respectively originated from distal bile duct (14 cases), ampulla (13 cases) and pancreas (73 cases). The median time from initial AP to tumor diagnosis was 8.57 weeks and the median number of episode was 2 in the tumor group, which significantly surpassed the non-tumor group (*p* = 0.000). Age, white blood cell count, percentage of neutrophils, pancreatic or bile duct dilation and recurrent attacks were selected independent predictors for tumor origin. A nomogram model based on these factors was established.

**Conclusion:**

For patients with agnogenic AP, elderly man, recurrent attacks, pancreatic or bile duct dilatation and continuous no significant increase of inflammatory markers prompt to further screening of pancreatic biliary and ampulla.

## Introduction

Acute pancreatitis (AP) is the most common principal gastrointestinal discharge diagnosis [1], with the year global incidence of 34/100000 [2]. The occurrence of an acute inflammatory process of the pancreatic parenchyma induced by mass of tumor has been reported [3]. As a doubling in the global annual number of pancreatic cancer diagnosed in the past two decades [4], tumor such as pancreatic cancer is becoming an increasing problem of pancreatitis-related hospitalization. However, international consensus guidelines on the diagnosis and management of tumor-induced AP do not yet specifically exist, and the effects of clinical characteristic and pathogenesis of tumor-induced AP on prognostic evaluation still remain controversial.

Tumor is easy to be misdiagnosed at the first admission due to the similar clinical characteristics of AP caused by whichever etiology [3]. It is a challenging dilemma, and meant to increase times and length of hospitalization, lead to serious complications [5], or even shorten the survival of AP patients. Significantly, there has been a growing appreciation of tumor-induced AP in recent years. It is crucial to timely identify tumor as the cause of this entity, and initiate a tailored treatment plan. In the present study, a reliable clinical prediction model was established by three machine learning algorithms. Multiple logistic regression model was developed to verify these predictors. In this way, we seek to highlight the clinical characteristics and pathogenesis on diagnosis and treatment of tumor-induced AP.

## Methods

### Patients selection

The data for the prevalence of tumor-induced AP came from retrospective studies. Patients firstly diagnosed AP at the First Affiliated Hospital of Zhengzhou University between 1 January 2013 and 31 December 2021 were enrolled in this study. Clinical medical information was obtained at the admission when AP and tumor diagnosis synchronously happened from the electronic medical record management system. AP patients induced by tumor were pathologically confirmed by histological examination of surgical specimens or fine needle biopsy, which were the tumor group. In contrast, 360 cases were random sampled from those AP patients with other etiologies using R software and the sample was used as the non-tumor group. Given the different primary origins, the tumor group was subdivided into the biliary group, the pancreas group and the ampulla group. Exclusions were made for the whole individuals with: (1) incomplete electronic medical records; (2) under 14 years old; (3) pregnant woman; (4) pancreatic and ampullary surgery and (5) history of pre-existing chronic pancreatitis; exclusions for the tumor group: (1) patients with incomplete pathological diagnosis information; (2) metastatic tumor. The follow-up was calculated from the date of histological diagnosis to 31 January 2022 or death. The study was approved by Ethical Committee of the First Affiliated Hospital of Zhengzhou University (2022-KY-0134).

### Diagnostic criteria

AP can be diagnosed if at least two of the following three criteria were fulfilled: (1) abdominal pain (acute onset of persistent and severe epigastric pain, often radiating to the back); (2) serum lipase (or amylase) activity at least three times the upper limit of normal; (3) or characteristic findings of AP on contrast-enhanced CT or, less often, MRI or transabdominal ultrasonography [6]. Severity of AP was classified into MAP, MSAP and SAP, based on the Revised Atlanta classification [7]. Recurrent acute pancreatitis was defined as one or more new episodes of acute pancreatitis at least 3 months after the first episode [8] and absence of morphological criteria for chronic pancreatitis [9]. The numerical rating scale (NRS) was used to assess pain. The TNM stage was evaluated according to the American Joint Commission on Cancer (8th Edition).

Pancreatic duct dilation was defined as the maximum diameter was greater than 3 mm, meanwhile, bile duct dilation was considered as the maximum diameter was greater than 7 mm [10]. Vascular invasion on CT was defined as the fat disappears between the mass and adjacent vessels, the mass wrapped around the adjacent vessel at > 180° and the presence of blocked blood vessels and stenosis [11]. All imaging studies were interpreted by two experienced radiologists who were unaware of the assignments.

### Statistical analysis

After filtering the data based on the exclusion criteria, percentages of tumor-induced AP among AP were annually calculated from 2013 to 2021. The etiological composition of patients with AP from 2019 to 2021 was further analyzed by horizontally comparing tumor to other etiologies.

The demographic differences of patients in tumor group were compared to those in non-tumor group. The LASSO regression model with the “lambda.1se” criterion, XGB and random forest were used for the selection of predictive variables. A 75:25 training and test split was used to train and evaluate independent predictors for tumor-induced AP. The randomized data division was developed to avoid data bias and ensure a balanced distribution in both the training and test sets. Performance of these multivariable models was evaluated with receiver-operating characteristic curves (ROC) and calibration. Multiple logistic regression analyses were used to calculate Relative Risk (RR) and corresponding 95% confidence intervals (CIs). A nomogram model was built to evaluate the probability of tumor-induced AP based on the selected predictors. Additional analyses were performed in individuals sorted by the different tumor locations to summarize the differences.

Quantitative variables were presented as mean ± standard deviation (SD) for normal distribution and median (*P*_25_–*P*_75_) for non-normal distribution. Categorical variables were presented as absolute numbers and percentages. *t*-test was used for continuously normally distributed variables of two groups, one-way ANOVA was used among three groups and SNK method was used for further pair comparison. Wilcoxon test was used to evaluate non-normally distributed quantitative variables of two groups, Kruskal–Wallis H test was used among three groups and Wilcoxon test was used for further pair comparison. Bonferroni was used to correct *P*. Chi-square test was performed on categorical variables. The cut-off value of the intervals from first episode of AP to tumor diagnosis was defined as the Optimal Operating Point of the ROC. The tumor specific survival curve was drawn using Kaplan–Meier method and assessed using the log-rank test. Statistical significance was set at a two-sided *p*-value equal to 0.05. All statistical analyses were performed by using SPSS software version 25 (IBM, USA, Version 22.0) and R software (University of Auckland, New Zealand, Version 4.1.1).

## Results

### Epidemiology

A total amount of 8970 patients were admitted for AP during the study period, and 8637 AP patients were enrolled in the study. The incidence of tumor-induced AP was 1.16% (100/8637), and the incidence was increasing year by year. Up to now, the annual incidence rates of tumor-induced AP from 2013 to 2021 were 0.29% (2/680), 0.14% (1/713), 0.25% (2/805), 0.23% (2/852), 0.46% (5/1090), 0.34% (4/1187), 0.81% (8/983), 3.82% (40/1046) and 2.81% (36/1281).

Among 3310 patients with AP from 2019 to 2021, miscellaneous causes included cholelithiasis (52.5%), hypertriglyceridemia (16.7%), idiopathic (12.7%), diet-related (9.5%), and infection (0.8%), alcohol (1.4%), autoimmune (0.4%), operation (1.5%), dialysis (0.1%), trauma (0.5%), congenital (0.1%), thrombosis (0.0%; 2 cases), pancreatolithiasis (0.5%), poisoning (0.2%), drugs (0.1%), tumor (2.4%), metastasis (0.2%), type 1 diabetes (0.0%; 2 cases), friction of drainage tube (0.0%; 2 cases), ileus (0.0%; 1 case), biliary hemorrhage after cholecystectomy (0.0%; 1 case) and familial adenomatous polyposis (0.0%; 2 cases).

### Baseline clinical features

In total, 100 individuals in tumor group and 360 admissions in the non-tumor group were enrolled (mean age 56.67 ± 11.03 versus 56.36 ± 10.70 years, *p* = 0.000) (Table 1). Compared with the control group, the tumor group was less likely to have gallstone-related episodes as the past history. More individuals in the tumor group had multiple episodes of AP than those in the non-tumor group (59% vs 13.3%, *p* = 0.000). More frequent attacks of AP were in the tumor group than the control group (Z = 8.403, *p* = 0.000). It took 8.57 (4.29 20.36) weeks from the first episode of AP to tumor diagnosis. The diagnosis rates at 1, 2, 3 months after the first admission were 37%, 58% and 70%. When comparing to the non-tumor group, the length of stay in the tumor group was longer (Z = 5.275, *p* = 0.005) and the median cost during the hospitalization was more (Z = 5.474, *p* = 0.001), respectively.

**Table 1:** Distribution of baseline characteristics in the tumor group and in the non-tumor group

Variables	Tumor *N* = 100	Non-tumor *N* = 360		*P*
Age (years)	56.67 ± 11.03	47.03 ± 15.32	*t* = 7.036	0.000
*Sex*				
Male	65 (65.0%)	237 (65.8%)	*χ*^*2*^ = 0.024	0.877
Female	35 (35.0%)	123 (34.2%)		
*Smoking*				
No	69 (69.0%)	270 (75%)	*χ*^*2*^ = 1.453	0.228
Yes	31 (31.0%)	90 (25%)		
*Alcohol consumption*				
No	84 (84.0%)	291 (80.8%)	*χ*^*2*^ = 0.521	0.470
Yes	16 (16.0%)	69 (19.2%)		
*Diabetes mellites*				
No	88 (88.0%)	310 (86.1%)	*χ*^*2*^ = 0.311	0.577
Yes	12 (12.0%)	50 (13.9%)		
*Cholelithiasis*				
No	74 (74.0%)	231 (64.2%)	*χ*^*2*^ = 3.387	0.066
Yes	26 (26.0%)	129 (35.8%)		
*Recurrent attacks*				
No	41 (41.0%)	312 (86.7%)	*χ*^*2*^ = 91.432	0.000
Yes	59 (59.0%)	48 (13.3%)		
*Pancreatic duct dilation*				
No	31 (31.0%)	316 (87.8%)	*χ*^*2*^ = 157.330	0.000
Yes	66 (66.0%)	30 (8.3%)		
Unknown	3 (3.0%)	14 (3.9%)		
*Bile duct dilation*				
No	36 (36.0%)	320 (88.9%)	*χ*^*2*^ = 149.367	0.000
Yes	62 (62.0%)	26 (7.2%)		
Unknown	2 (2.0%)	14 (3.9%)		
*Severity of pancreatitis*				
Mild	90 (90.0%)	208 (57.8%)	*H* = 35.626	0.000
Moderately severe	9 (90.%)	134 (37.2%)		
Severe	1 (1.0%)	18 (5.0%)		
White blood cells (× 10^9^/L)	6.00 (4.63 7.78)	10.00 (6.95 14.30)	*Z* = 7.811	0.000
Hemoglobin (g/L)	127.00 (118.00 137.85)	131.00 (116.00 148.00)	*Z* = 2.082	0.037
Hematocrit value	0.39 (0.36 0.42)	0.40 (0.35 0.44)	*Z* = 1.634	0.102
Neutrophilic granulocyte percentage (%)	67.7 (58.4 73.6)	81.6 (69.9 88.3)	*Z* = 7.735	0.000
Albumin (g/L)	39.7 (36.7 42.2)	38.1 (33.4 41.7)	*Z* = 2.749	0.006
Alkaline phosphatase (U/L)	132 (83 298)	82 (64 126)	*Z* = 5.755	0.000
Direct bilirubin (μmol/L)	8.7 (4.3 94.4)	7.1 (4.4 16.0)	*Z* = 2.634	0.008
Indirect bilirubin (μmol/L)	7.0 (4.2 15.6)	7.4 (4.9 13.3)	*Z* = 0.403	0.687
Creatinine (μmol/L)	64 (55 78)	64 (52 80)	*Z* = 0.161	0.872
Amylase (U/L)	145 (73 275)	155 (75 475)	*Z* = 1.228	0.219
Lipase (U/L)	303.0 (181.7 874.7)	201.3 (80.9 624.5)	*Z* = 3.351	0.001
C-reactive protein (mg/L)	5.39 (1.82 35.45)	51.10 (9.76 159.35)	*Z* = 4.552	0.000
Serum glucose (mmol/L)	5.95 (4.64 7.47)	6.86 (5.20 9.32)	*Z* = 2.958	0.003
Serum calcium (mmol/L)	2.29 (2.21 2.35)	2.18 (2.06 2.29)	*Z* = 5.875	0.000
Triglyceride (mmol/L)	1.31 (0.84 2.11)	1.75 (0.94 4.40)	*Z* = 2.919	0.004
Time (day)	19 (12 29)	11 (8 18)	*Z* = 5.877	0.000
Cost (yuan)	64,297.80 (21,911.23 108,028.30)	23,454.58 (14,498.46 43,762.65)	*Z* = 6.209	0.000

In tumor group, progressively aggravated intense pain in the upper abdomen was special symptom of 88 patients. Of them, 23 (26.1%) patients had previous attacks of pancreatic pain before first admission. Obstructive jaundice was in 14 patients which may result from tumor itself. MAP was observed in 90 cases, MSAP in 9 cases and SAP in one case.

87 patients (87%) underwent computed tomography (CT), 69 patients (69%) underwent ultrasound (US), 47 patients (47%) underwent magnetic resonance cholangiopancreatography (MRCP), 9 patients (9%) underwent endoscopic ultrasonography (EUS) and 11 patients (11%) underwent positron emission tomography-computed tomography (PET-CT). The sensitivities of CT, US, MRCP, EUS and PET-CT were 97.7%(85/87), 84.1%(58/69), 95.7%(45/47), 100%(9/9) and 81.8%(9/11), which assessed the rate of the number of tumors detected by imaging modalities to the number of exact cases. The presentation under CT was diffuse or focal volume enlargement of the pancreas and diffuse, segmental and cystic expansion of the pancreatic ducts. US showed diffuse enlargement of pancreatic volume, reduced internal echo and unclear boundary. The sensitivity and specificity of pancreatic duct dilation were 68.0% and 91.3%. And the sensitivity and specificity of bile duct dilation were 63.3% and 92.5%. The sensitivity and specificity of double duct sign (DDS) which represented co-existence of pancreatic and bile duct dilation were 51.0% and 96.5%.

### Baseline parameters of patients with different tumors

There were 73 cases located in pancreas, 13 cases in ampulla and 14 cases in extrahepatic bile duct. The clinical characteristics of patients with tumor in different sites were summarized in Table 2. Table 3 summarized the indexes with statistically significant differences by pairwise comparison. Pancreatic duct dilation was more likely to be seen in the pancreas group than the ampulla group. The sizes of the pancreas group were significantly larger than those of the biliary and ampulla group. Albumin in the pancreas group was higher than the biliary group and CEA in the pancreas group was higher than the ampulla group (all *p* < 0.05).

**Table 2:** Clinicopathological features of tumor-induced AP in different sites

Variables	Pancreas *N* = 73	Ampulla *N* = 13	Biliary tract *N* = 14		*P*
Age (year)	57.15 ± 11.52	54.31 ± 8.71	56.36 ± 10.70	*F* = 0.369	0.693
Sex				*χ*^*2*^ = 0.16	0.945
Male	47 (64.4%)	9 (69.2%)	9 (64.3%)		
Female	26 (35.6%)	4 (30.8%)	5 (35.7%)		
Severity of pancreatitis				*χ*^*2*^ = 0.801	0.747
Mild	66 (90.4%)	11 (84.6%)	13 (92.9%)		
Moderate severe/severe	7 (9.6%)	2 (15.4%)	1 (7.1%)		
Recurrent attacks				*χ*^*2*^ = 1.754	0.416
No	28 (38.4%)	5 (38.5%)	8 (57.1%)		
Yes	45 (61.6%)	8 (61.5%)	6 (42.9%)		
Pancreatic duct dilation				*χ*^*2*^ = 10.131	0.025
No	16 (21.9%)	8 (61.5%)	7 (50.0%)		
Yes	54 (74.0%)	5 (38.5%)	7 (50.0%)		
Unknown	3 (4.1%)	0	0		
Bile duct dilation				*χ*^*2*^ = 2.662	0.633
No	25 (34.2%)	7 (53.8%)	4 (28.6%)		
Yes	46 (63.0%)	6 (46.2%)	10 (71.4%)		
Unknown	2 (2.7%)	0	0		
Diameter of tumor (cm)	4.0 (2.3 4.8)	1.8 (1.5 2.5)	1.7 (1.5 2.5)	*H* = 17.602	0.000
White blood cells (× 10^9^/L)	5.41 (4.52 7.54)	5.91 (4.98 7.15)	7.53 (5.67 8.03)	*H* = 2.451	0.294
Hemoglobin (g/L)	127.02 ± 15.06	123.74 ± 20.10	125.06 ± 14.55	*F* = 0.264	0.768
Neutrophilic granulocyte percentage (%)	66.54 ± 11.68	58.25 ± 22.74	65.22 ± 10.04	*F* = 1.892	0.157
Hematocrit value	0.386 ± 0.044	0.370 ± 0.060	0.372 ± 0.047	*F* = 1.007	0.369
Amylase (U/L)	138.0 (77.5 234.5)	166.0 (72.0 949.0)	245.0 (48.0 528.0)	*H* = 1.981	0.371
Lipase (U/L)	303.00 (205.10 862.20)	275.35 (131.55 1434.28)	309.15 (137.63 1165.00)	*H* = 0.250	0.883
Serum glucose (mmol/L)	6.03 (4.98 7.89)	4.64 (4.19 6.01)	6.20 (5.02 10.29)	*H* = 4.873	0.087
Serum calcium (mmol/L)	2.30 (2.24 2.35)	2.22 (2.13 2.44)	2.23 (2.09 2.32)	*H* = 5.651	0.059
Albumin (g/L)	40.03 ± 4.23	41.42 ± 8.92	36.05 ± 4.42	*F* = 4.506	0.014
Direct bilirubin (μmol/L)	8.15 (4.16 110.60)	6.75 (3.63 49.18)	26.40 (6.20 137.95)	*H* = 2.035	0.362
Indirect bilirubin (μmol/L)	7.35 (4.38 18.70)	4.40 (2.80 9.20)	5.80 (2.85 20.20)	*H* = 3.793	0.15
CEA (ng/ml)	4.06 (2.11 8.50)	1.48 (0.68 2.39)	2.75 (1.14 5.37)	*H* = 12.822	0.002
CA125 (U/ml)	23.47 (15.45 91.22)	38.30 (13.18 272.19)	23.15 (8.93 43.65)	*H* = 1.800	0.407
CA199 (U/ml)	193.30 (48.12 1601.50)	69.83 (6.86 322.25)	131.00 (22.64 411.00)	*H* = 5.258	0.072
Stage				*χ*^*2*^ = 34.034	0.000
I	17 (23.3%)	3 (23.1%)	1 (7.1%)		
II	10 (13.7%)	1 (7.7%)	6 (42.9%)		
III	19 (26.0%)	0	3 (21.4%)		
IV	27 (37.0%)	3 (23.1%)	2 (14.3%)		

*CEA*, carcino-embryonic antigen; *CA125*, carbohydrate antigen 125; *CA199*, carbohydrate antigen 199

**Table 3:** Pairwise comparison of characteristics of different origin tumor

Variables	Pancreas versus ampulla		Pancreas versus biliary tract		Ampulla versus biliary tract	
		*p*		*p*		*p*
Pancreatic duct dilation		< 0.05		> 0.05		> 0.05
Diameter of tumor (cm)	*H*=2.675	0.022	*H*=3.629	0.001	*H*=0.053	1.000
Albumin (g/L)	*F*=0.871	1.000	*F*=2.933	0.010	*F*=2.053	0.120
CEA (ng/ml)	*H*=3.355	0.002	*H*=1.697	0.269	*H*=1.476	0.420

*CEA*, carcino-embryonic antigen

In the pancreas group, tumors mostly located in head and neck (56/73) while partly in body and tail (17/73). The comparisons of tumor characteristics in different parts of pancreas were shown in Table 4*.* According to pathological classification, there were 59 adenocarcinomas, 7 mucinous cystadenomas, 2 acinar cell carcinomas, 1 squamous cell carcinoma, 2 small cell carcinomas, 1 solid pseudopapilloma and 1 intraductal papilloma. Vascular invasion was observed in 14 cases. 5 cases synchronously violated portal vein and mesenteric vessels, 1 case synchronously violated liver and pancreas itself (body and tail) as well as 1 case synchronously violated psoas major and ilium. Single organ metastases included liver (17 cases), duodenum (3 cases), bone (1 case) and common bile duct (1 case). There are 27 cases (37.0%) in stage IV, 19 cases (26.0%) in stage III, 10 cases (13.7%) in stage II (3 case stage IIB and 7 case stage IIA), 17 cases (23.3%) in stage I (14 case stage IB and 3 case stage IA).

**Table 4:** Demographics and clinical characteristics of patients with pancreatic tumor-induced AP

Variables	Head and neck *N* = 56	Body and tail *N* = 17		*P*
Age (year)	58.36 ± 10.79	53.18 ± 13.23	*t* = 1.644	0.105
Sex			*χ*^*2*^ = 5.205	0.041
Male	40 (71.4%)	7 (41.2%)		
Female	16 (28.6%)	10 (58.8%)		
Pancreatic duct dilation			*χ*^2^=18.423	0.000
No	6 (10.7%)	10 (58.8%)		
Yes	48 (85.7%)	6 (35.3%)		
Unknown	2 (3.6%)	1 (5.9%)		
Bile duct dilation			*χ*^2^=19.570	0.000
No	12 (21.4%)	13 (76.5%)		
Yes	43 (76.8%)	3 (17.6%)		
Unknown	1 (1.8%)	1 (5.9%)		
Pathological classification			χ^2^=19.473	0.000
Adenocarcinoma	51 (91.1%)	8 (47.1%)		
Solid-pseudopapillary tumor	0	1 (5.9%)		
Small cell carcinoma	0	2 (11.8%)		
Mucinous neoplasms	3 (5.4%)	4 (23.5%)		
Squamous-cell carcinoma	1 (1.8%)	0		
Intraductal papilloma	0	1 (5.9%)		
Acinar cell carcinoma	1 (1.8%)	1 (6.3%)		
Diameter of tumor (cm)	4.00 (2.15 4.63)	3.00 (2.40 5.50)	*Z* = 0.333	0.749

In the ampulla group, there were 6 villous tubular adenomas and 7 adenocarcinomas. Liver metastasis was observed in 1 case and biliary duct was involved in two cases. There were 3 cases (23.1%) in stage IV, 1 case (7.7%) in stage IIB and 3 cases (23.1%) in stage IB. In the biliary group, there were 12 adenocarcinomas, 1 high-grade neoplasia with canceration and 1 villous adenoma with canceration. Vascular invasion was observed in 2 cases. Single organ metastases included pancreas (1 case), duodenum (1 case) and liver (2 cases). And there were 2 cases (14.3%) in stage IV, 3 cases (21.4%) in stage III, 6 cases (42.9%) in stage II (3 case stage IIB and 3 case stage IIA) and 1 case (7.1%) in stage IB.

Totally, 32% cases were IV stage, 22% cases were III stage, 17% cases were II stage (7 case IIB and 10 case IIA) and 21% cases were I stage (18 case IB and 3 case IA). The differences among the three groups were statistically significant (*p* = 0.000).

### Predictors for tumor-induced AP

**Fig. 1:**
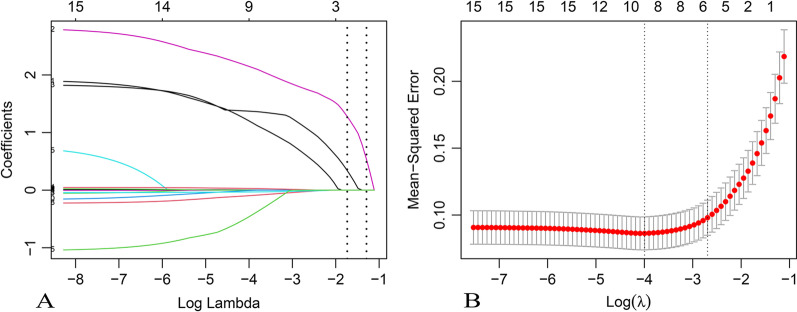
Selection of risk factors of tumor-induced AP using the LASSO logistic regression algorithm. **A** LASSO coefficient profiles of the 15 candidate variables. Vertical line was plotted at the given lambda, selected by tenfold cross-validation with minimum classification error and minimum classification error plus 1 standard error, respectively. For the optimal lambda that gives minimum classification error plus 1 standard error, 6 features with a non-0 coefficient were selected. **B** Penalization coefficient lambda in the LASSO model was tuned using tenfold cross-validation and the “lambda.1se” criterion. Area under the curve (AUC) metrics (y-axis) were plotted against log (lambda) (bottom x-axis). Top x-axis indicates the number of predictors for the given log (lambda). Red dots indicate average AUC for each model at the given lambda, and vertical bars through the red dots show the upper and lower values of the AUC according to the 10-fold cross-validation. Vertical black lines define the optimal lambda that gives the minimum classification error plus 1 standard error

**Fig. 2:**
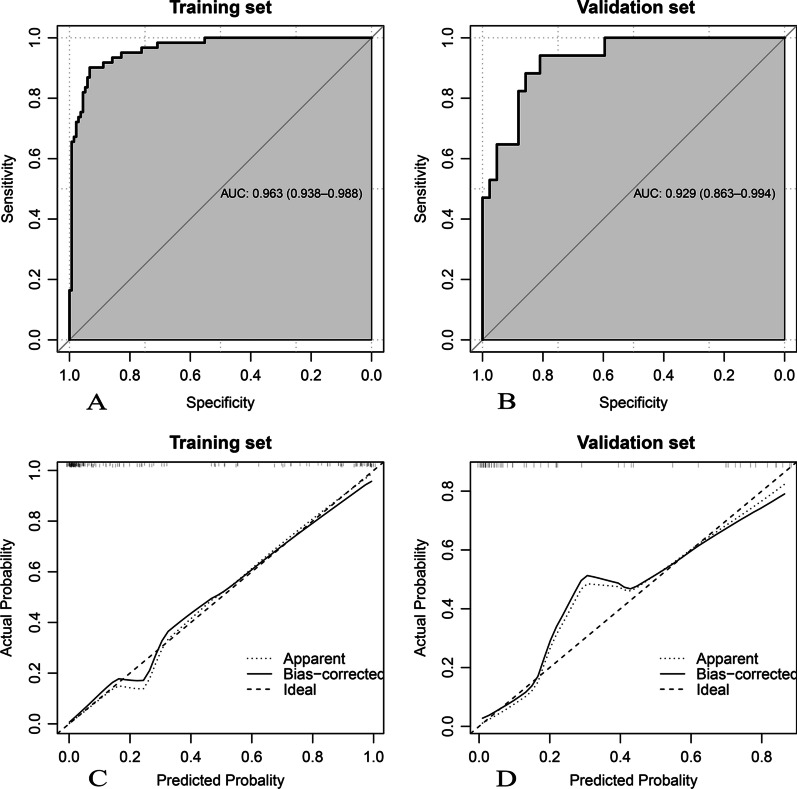
Performance of the logistic regression algorithm in tumor-induced AP prediction. (**A**, **B**) Receiver-operating characteristic curves; (**C**, **D**) calibration curves

**Fig. 3:**
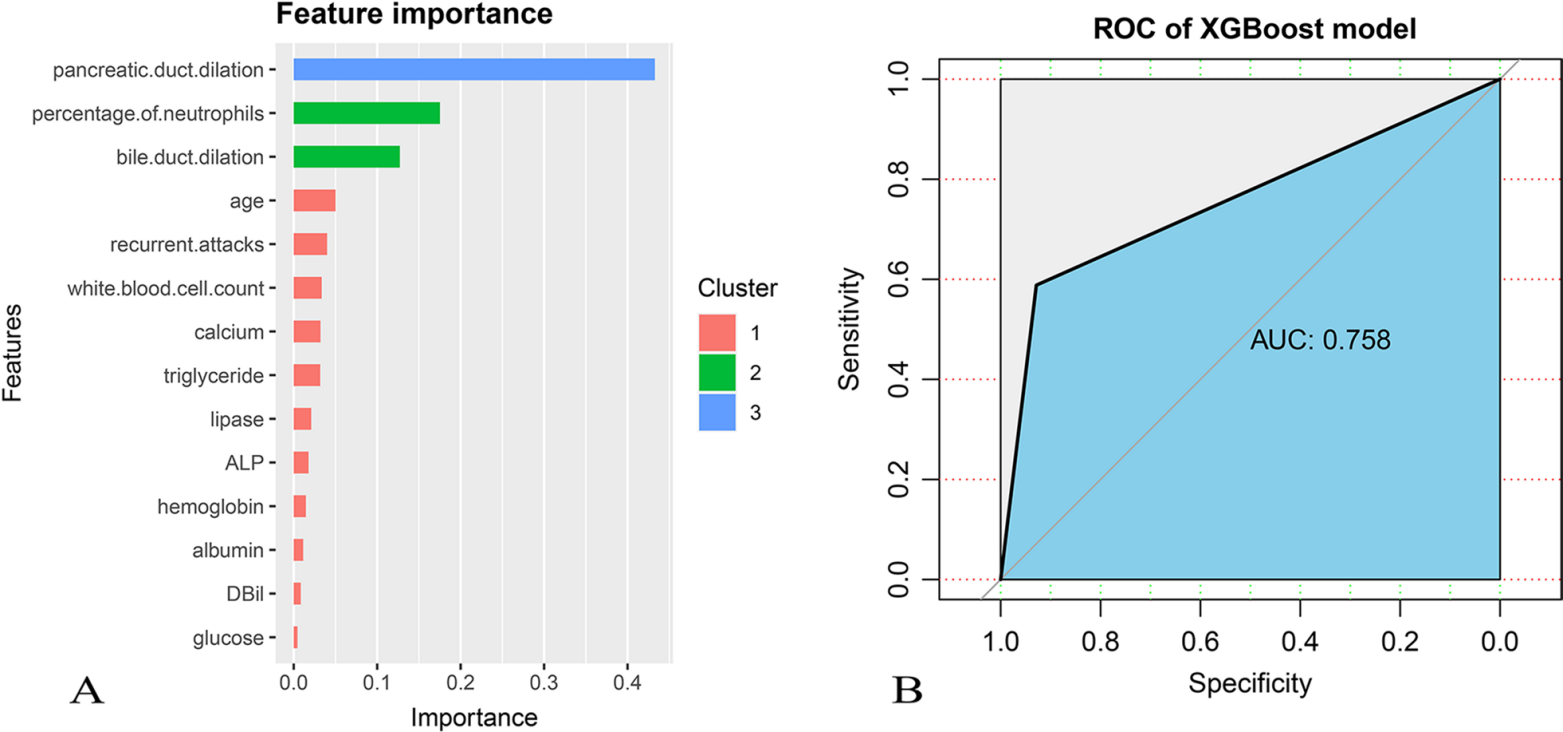
**A** Feature correlations and distributions of feature importance for the XGBoost model. Model input variables are ranked in descending order of feature importance. **B** The receiver operating characteristic curve was plotted though the test set evaluated the trained model. ALP: alkaline phosphatase; DBil: direct bilirubin

**Fig. 4:**
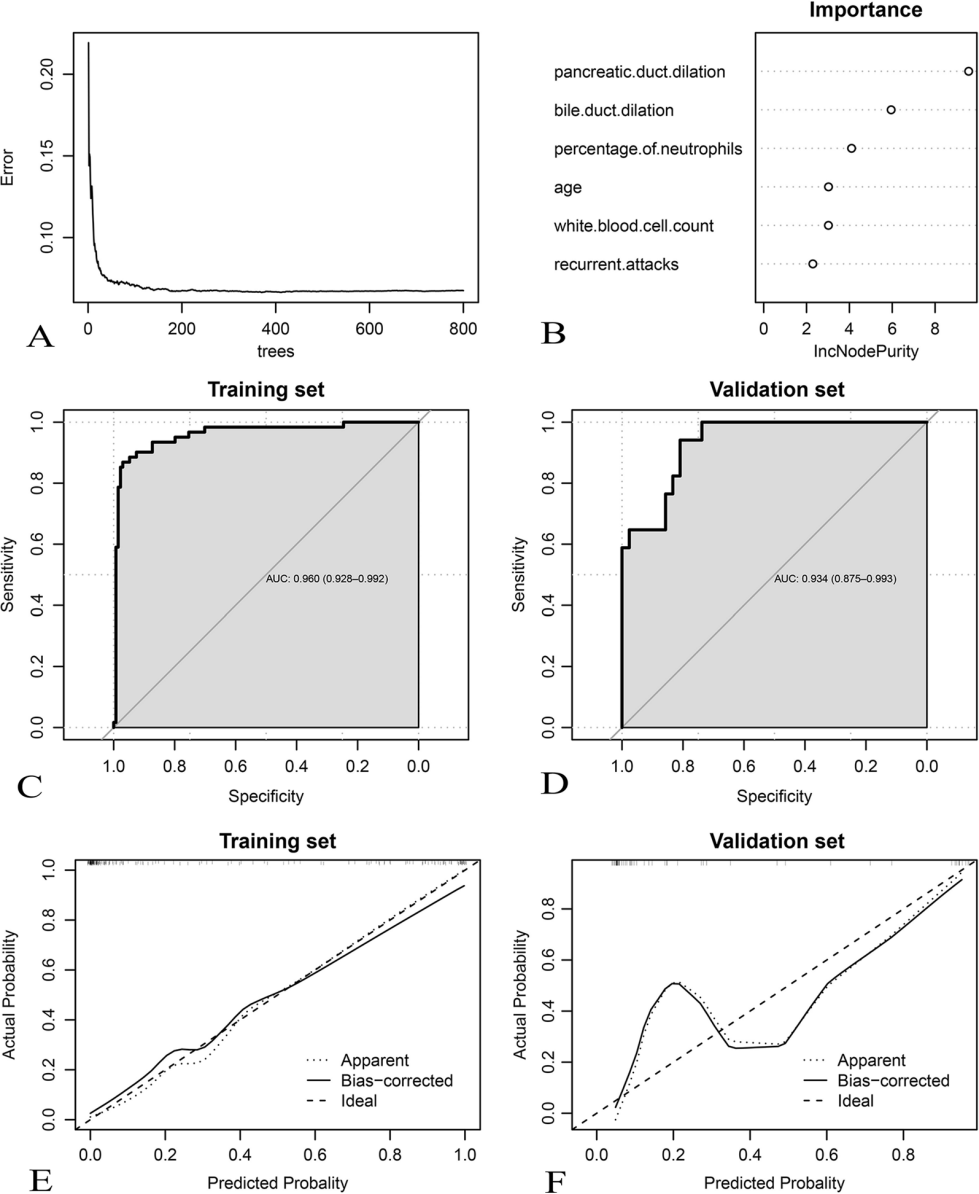
Development and assessment of the random forest algorithm in tumor-induced AP prediction. **A** Relationship between out-of-bag error and number of trees. In total, 139 trees are selected to establish a random forest model; **B** feature importance; (**C**, **D**) Receiver-operating characteristic curves; (**E**, **F**) calibration curves

**Fig. 5:**
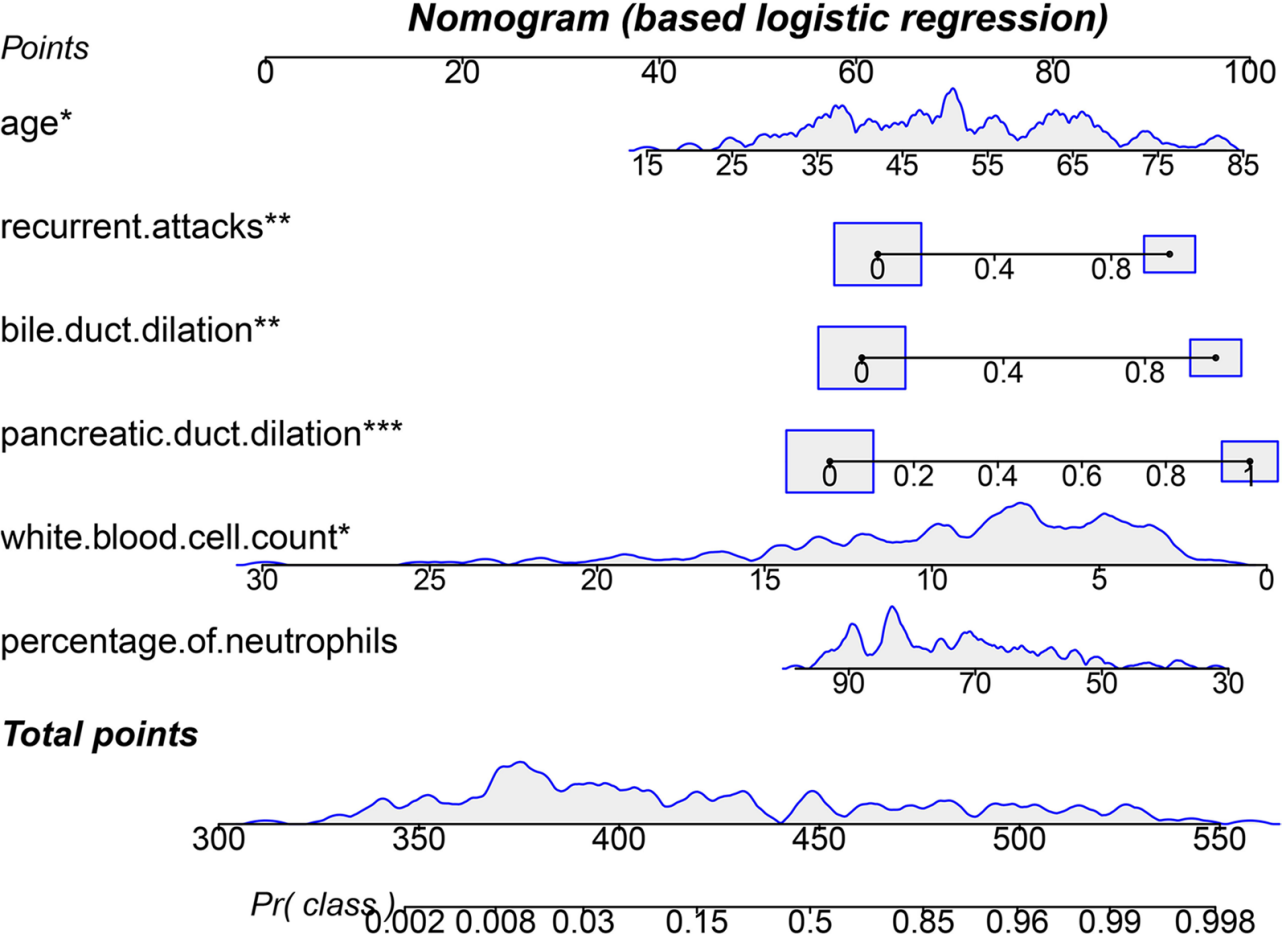
Nomogram of tumor-induced AP prediction was developed using the multivariate logistic regression model. Value assigned to each factor was scored on a scale of 0–100. By adding scores for each factor, one can obtain a total score. On the basis of the total score, the probability of tumor-induced AP is displayed by projecting the score to the bottom risk axis. *: *p* < 0.05; **: *p* < 0.01; ***: *p* < 0.001

All available features with statistical significance in Table 1 except C-reactive protein (CRP) which had many missing values, cost and time were trained by LASSO regression (Figs. 1, 2), XGB (Fig. 3) and random forest model (Fig. 4). Calibration plots and receiver operator characteristic curves were used to evaluate the model. Interestingly, all three methods revealed that age, recurrent attacks, pancreatic duct dilation, bile duct dilation, white blood cell count and percentage of neutrophils were predictors of tumor-induced AP. These variables, as age (1.05 [1.01 1.10], *p* = 0.013), recurrent attacks (6.19 [1.91 22.05], *p* = 0.003), pancreatic duct dilation (13.74 [4.04 53.29], *p* = 0.000), bile duct dilation (8.99 [2.42 36.57], *p* = 0.001), white blood cell count (0.81 [0.66 0.97], *p* = 0.032) and percentage of neutrophils (0.96 [0.92 1.00], *p* = 0.108) were calculated in logistic regression analysis and then used to develop a nomogram (Fig. 5).

### Treatment and survival analysis

Endoscopic ultrasound-guided fine-needle biopsy (EUS-FNA) has performed in 7 patients. 38 patients underwent CT-guided biopsy to confirm the diagnosis. 41 patients were managed with pancreaticoduodenectomy, 13 patients underwent distal pancreatectomy, 2 underwent endoscopic ultrasound and endoscopic resection, 1 underwent endoscopic resection and stent implantation, 1 underwent nasojejunal feeding tube implement under endoscopy, 2 underwent cholangiography and drainage tube placement and 1 underwent percutaneous transhepatic cholangial drainage (PTCD). 7 patients merely received chemotherapy. 14 patients received surgical procedures combined with systemic chemotherapy.

**Fig. 6:**
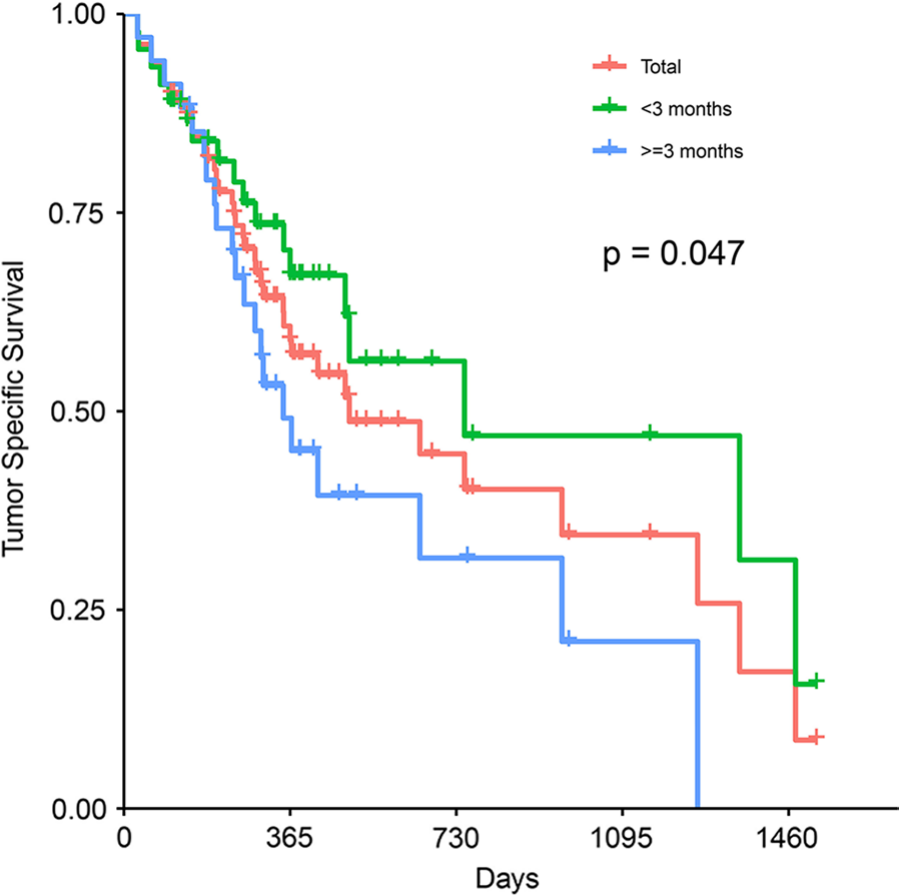
Kaplan–Meier curve for tumor specific survival of patients by whether the time from first attack of AP to tumor diagnosis was longer than 3 months or not

There was significantly lower 3 year survival rate for no less than 3 months from AP onset to tumor diagnosis than < 3 months (*p* = 0.047; Fig. 6). The 1, 2, 3 and 4 year cumulative survival rates of patients with tumor-induced AP were 58.9%, 44.6%, 34.4% and 17.2%. The median overall survival was 495 days (interquartile range 190.12–799.88 days).

## Discussion

Correctly diagnosing AP etiology is a crucial step to prescribe the most appropriate therapy and prevent recurrent attacks [12].The distribution of etiologies of AP differs by (1) region, with gallstone and alcohol as the leading causes in the United States and a predominance of gallstones in southern Europe [13], (2) period, with increased non-gallstone-related AP incidence in the early 1990s and decreased incidence in the mid-1990s to mid-2000s [14]. It is noted that tumor as currently common etiology of AP patients in China is increasingly recognized. As the overlap in clinical and imaging features of tumor-induced AP and AP from other etiologies, controversy regarding tumor-induced AP diagnosis (including clinical symptoms, biochemical and morphologic) renders differential diagnosis challenging. For comparison against the LASSO regression, more structurally complex XGB and Random forest were used to investigate predictors of tumor-induced AP in the single center cohort. A new nomogram model that consisted of age, white blood cell count, percentage of neutrophils, pancreatic or bile duct dilation and recurrent attacks was built to create a high predictive value.

A series of events with regard to mass, ranging from pancreatic-duct obstruction, and/or stasis of pancreatic juice, appear to play a fundamental role in the pathogenesis of tumor-induced AP. Mujica et al. also speculated these pathophysiological events may trigger a series of cascading events causing acinar cell disruption, intracellular activation of proteolytic enzymes, pancreatic parenchymal edema, and peripancreatic inflammation and even pancreatic enzymes were activated directly by tumor tissue [15]. Compared with previous studies [16, 17], this study concentrated on three anatomically adjacent locations, pancreas, the papilla Vater and distal bile duct, to assess whether the tumor location influenced the differences in pathogenesis. Pancreas was the main tumor location, especially the head and neck of pancreas, followed by distal bile duct and ampulla. Among the tumors from different sites, pancreatic tumor diameter was significantly larger than the other two groups, suggesting that bile tract and ampullary tumor may have a higher rate of misdiagnosis, and need close follow-up and comprehensive imaging monitor.

With pathology providing the most robust evidence to confirm the etiology, adenocarcinoma, adenoma and mucinous tumor has become common histological types of tumor with recurrent AP as the initial symptom. Squamous cell carcinoma [18], non-Hodgkin’s lymphoma [19] and adenomyoma [20] can also be seen. In this study, both benign and malignant tumor associated AP could induce abdominal effusion, but malignant ascites was mostly related to metastasis. Previous studies have reported that ascites with high amylase content was usually associated with benign tumors [21]. Therefore, ascites biochemical and abscisic cytology examination are of great significance for the comprehensive diagnosis of AP.

Smoking, alcohol consumption and diabetes were shared risk factors of AP and tumor [22], however, there was no significant difference between two groups. Notably, patients with tumor-induced AP were more likely to be older, male and mild entity compared to AP patients with other etiologies. The tumor group mostly presented with intermittently progressive epigastric pain which may result in the difficulty for individual first diagnosis. Furthermore, the time from initial AP to tumor diagnosis seemed to be delayed as Omid et al. described [8] and there were more episodes compared to the non-tumor group. The factors that interfere with an early diagnosis and cause recurrent attacks include that a mild course [7], ineffective imaging screening to distinguish between inflammatory edema and tumorous mass [23] and not very valuable information associated with tumor-induced AP owing to low sensitivity. Both age and recurrent attacks were the independent predictors.

Most cases were MAP which was associated with absence of organ dysfunction [24] and local or systemic complications. The suspicion appeared strengthened when MAP was combined with lower infection index including the white blood cell count, percentage of neutrophils and serum CRP. The white blood cell count was proved to be an independent predictor in the model. Percentage of neutrophils was of importance in ensemble learning, but it was validated to be not significant in the multiple logistic regression (*p* = 0.108). The effect of percentage of neutrophils needs to be further explored.

In tumor group, lipase was significantly higher than that in the control group while amylase was not. The elevated serum lipase was also seen in pancreatic and ampullary acinar cell carcinoma [25, 26] and it was an additional tool for determinations of recurrence or treatment response as Kruger suggested [27]. Significantly elevated alkaline phosphatase and direct bilirubin have been associated with biliary obstruction in tumor group. There was a persistent increase trend in specific tumor makers, such as CEA, CA199, and CA125. The level of CEA in the pancreas group was higher than the ampulla group, which confirmed malignancies accounted for majority.

Our findings reinforce the widely held belief that precise imaging should be performed when the diagnosis is compounded and patients fail to clinically improve after 2–3 days [28, 29]. CT is widely performed for the assessment of AP severity, however, its contribution to the etiologic diagnosis should not be overlooked [30]. Cho et al. suggested that patients with idiopathic AP should complete CT within 3 months after discharge to exclude occult tumors [31]. When CT does not reassure us against occult malignancy, MRI and MRCP offer alternatives with higher sensitivity but more cost, and EUS has no false positive mass detections when compared with final histopathological diagnosis. Moreover, endoscopic retrograde cholangiopancreatography (ERCP) can diagnose intraductal lesions, relieve obstruction by placing a stent and prevent recurrence until an established diagnosis and more definitive treatment can be offered [32]. We believe that abdominal appearance on imaging should be incorporated into the decision algorithm for early diagnosis of tumor-induced AP.

Radiologic features include but are not limited to focal or diffuse volume enlargement, peripancreatic fluid collections. The classical features of tumor-induced AP involve pancreatic duct dilation [23]. What’s more, co-existence of pancreatic and bile duct dilation (DDS) can improve the diagnostic specificity. Both of them were independent predictors of tumor-induced AP. In this study, pancreatic duct dilation was more likely to occur in pancreatic tumor, especially the head and neck of pancreas. No difference of bile duct dilation was identified among the three groups. DDS is more common in pancreatic head-neck tumor than body-tail tumor as mass obstructs ducts causing marked dilation of upstream duct [17]. DDS has long been considered ominous given its presence in cases of periampullary malignancy. Of note, dilated pancreatic or bile duct in high risk patients with clinical signs require comprehensive workup and careful evaluation of the entire gland and surrounding abdominal structures [33].

When comparing to AP from other etiologies, tumors not only prolong hospitalization of AP patients, but also increase financial burden. The initial management of tumor-induced AP is similar to the others; nevertheless, exact clinical treatment has not been found to improve the whole disease course. Inflammation can increase the difficulty of surgery and postoperative complications by aggravating tissue adhesion and edema, thus affecting surgical efficacy. Timely control of inflammation progression is critical for subsequent treatment [34]. Pancreaticoduodenectomy is the key treatment for malignant tumors in this region [9]. Endoscopic treatment is feasible for small, benign tumors [9], or patients with greater surgical risk than benefit.

Earlier recognition of tumor after the first episode of AP is associated with a less advanced cancer and increases the probability of surgery with curative intent [8]. Patients had low prevalence of distant organ metastasis at diagnosis (26.2%) and high resection frequencies (60.7%) in this study. Although MAP was not generally associated with mortality [35], the 4-year cumulative survival rate of patients with tumor-induced AP was 17.2%. It may be related to the fact that most entities were aggressive (e.g. Stage III/IV tumors accounted for 54% of the patients) and partly preformed palliative. The survival analysis revealed that the outcomes of patients with an interval of less than 3 months between the first episode of AP and tumor diagnosis were more favorable than those of patients with an interval of more than 3 months. Based on poor prognosis of periampullary and pancreatic tumor-induced AP [36], pathogenesis contributes to determinate individualized management strategies and improve prognosis.

However, there are still some limitations to this study. First, it is a retrospective study that is unavoidable to be affected by selection bias. Second, the data, such as the stage about adenoma and high-grade neoplasia, was not included in the study. Third, since it is a single-centre study, it limits the generalization of our findings to other institutions or populations with different resources. Prospective, multicenter and larger scale researches are still needed to confirm the differences in survival outcomes which caused by the initial concerns. The present work supported the value of machine learning methods for exploring for etiology of AP. There remains significant room to develop, validate and implement machine learning based AP diagnostic tools in clinical settings.

## Conclusion

More cases of tumor-induced AP than expected have been confirmed, and more standardized guidelines for investigating tumor-induced AP should be put in place. Early elaborate examination of pancreas bile and ampulla is essential for tumor-induced AP. The elderly, recurrence attacks, white blood cell count, percentage of neutrophils and pancreatic or bile ducts dilatation are pretty reliable predictors for diagnosis of tumor-induced AP, thus they should be considered in etiology exploration and the ongoing individualized management of tumor-induced AP.

## Data Availability

The data that support the findings of this study are available from the corresponding author upon reasonable request.

## Abbreviations

AP: Acute pancreatitis
MAP: Mild acute pancreatitis
MSAP: Moderately severe acute pancreatitis
SAP: Severe acute pancreatitis
LASSO: Least absolute shrinkage and selection operator
XGB: XGBoost
CT: Computed tomography
MRI: Magnetic resonance imaging
MRCP: Magnetic resonance cholangiopancreatography
US: Transabdominal ultrasound
PET-CT: Positron emission tomography-computed tomography
ERCP: Endoscopic retrograde cholangiopancreatography
EUS: Endoscopic ultrasonography
PTCD: Percutaneous transhepatic cholangial drainage
ROC: Receiver-operating characteristic curves
RR: Relative risk
CI: Confidence interval
DDS: Double duct sign
CRP: C-reactive protein
CEA: Carcinoembryonic antigen
